# The Mechanism Underlying the Influence of Indole-3-Propionic Acid: A Relevance to Metabolic Disorders

**DOI:** 10.3389/fendo.2022.841703

**Published:** 2022-03-18

**Authors:** Binbin Zhang, Minjie Jiang, Jianan Zhao, Yu Song, Weidong Du, Junping Shi

**Affiliations:** ^1^Department of Translational Medicine Platform, The Affiliated Hospital of Hangzhou Normal University, Hangzhou, China; ^2^College of Life Sciences, Zhejiang University of Traditional Chinese Medicine, Hangzhou, China; ^3^Zhejiang University of Traditional Chinese Medicine, Hangzhou, China; ^4^Guanghua Clinical Medical College, Shanghai University of Traditional Chinese Medicine, Shanghai, China; ^5^Zhejiang Traditional Chinese Medicine Hospital, Hangzhou, China; ^6^Department of Infectious & Hepatology Diseases, Metabolic Disease Center, The Affiliated Hospital of Hangzhou Normal University, Hangzhou, China

**Keywords:** metabolic syndrome, indole-3-propanoic acid, obesity, type 2 diabetes, non-alcoholic fatty liver disease, cardiovascular diseases

## Abstract

The increasing prevalence of metabolic syndrome has become a serious public health problem. Certain bacteria-derived metabolites play a key role in maintaining human health by regulating the host metabolism. Recent evidence shows that indole-3-propionic acid content can be used to predict the occurrence and development of metabolic diseases. Supplementing indole-3-propionic acid can effectively improve metabolic disorders and is considered a promising metabolite. Therefore, this article systematically reviews the latest research on indole-3-propionic acid and elaborates its source of metabolism and its association with metabolic diseases. Indole-3-propionic acid can improve blood glucose and increase insulin sensitivity, inhibit liver lipid synthesis and inflammatory factors, correct intestinal microbial disorders, maintain the intestinal barrier, and suppress the intestinal immune response. The study of the mechanism of the metabolic benefits of indole-3-propionic acid is expected to be a potential compound for treating metabolic syndrome.

## Introduction

Metabolic syndrome is defined as a group of interrelated comprehensive diseases characterized by visceral obesity, hypertension, hyperlipidemia, atherosclerosis, and insulin resistance. Metabolic syndrome, including overweight/obesity, type 2 diabetes (T2D) (1, 2), non-alcoholic fatty liver disease (NAFLD) (3), and cardiovascular disease (CVD) (4) has become a severe public health problem (5). Accumulating evidence has linked intestinal microbe imbalance or compositional changes with the pathogenesis of metabolic diseases (6). Intestinal microbes produce functional metabolites that regulate intestinal endocrine function and neural signals, regulate energy metabolism, and affect host immune mechanisms and homeostasis (7). Functional metabolites serve as potential markers of disease and transfer to distant organs through the intestinal barrier–peripheral circulation, affecting the metabolic phenotype of the host (8–10). Therefore, the link between functional metabolites and metabolic diseases has received increasing attention.

Indole-3-propanoic acid (IPA) is a tryptophan (Trp) metabolite produced by intestinal bacteria that is closely associated with diet. IPA has received increasing attention in recent years because of its close correlation with metabolic diseases. Recent studies have found that IPA content can predict the occurrence of obesity (11), T2D (12), NAFLD (13), and CVD (14).

In recent years, supplementation with IPA has been shown to improve blood glucose, increase insulin sensitivity (15), inhibit liver lipid synthesis and inflammatory factors (16), correct intestinal microbial disorders (17), maintain the intestinal barrier, and suppress the intestinal immune response (18). Here, we systematically reviewed the latest research on IPA, its association with metabolic diseases, and its role in metabolic disorders, and discuss its future research directions.

## IPA Is the Metabolite of Trp in the Intestine

IPA is a metabolite produced by the microflora of dietary Trp that accumulates in the host serum and exhibits high individual differences (19). Under physiological conditions, serum IPA concentrations range from 1 to 10 µM in humans (20, 21)

**Figure 1:**
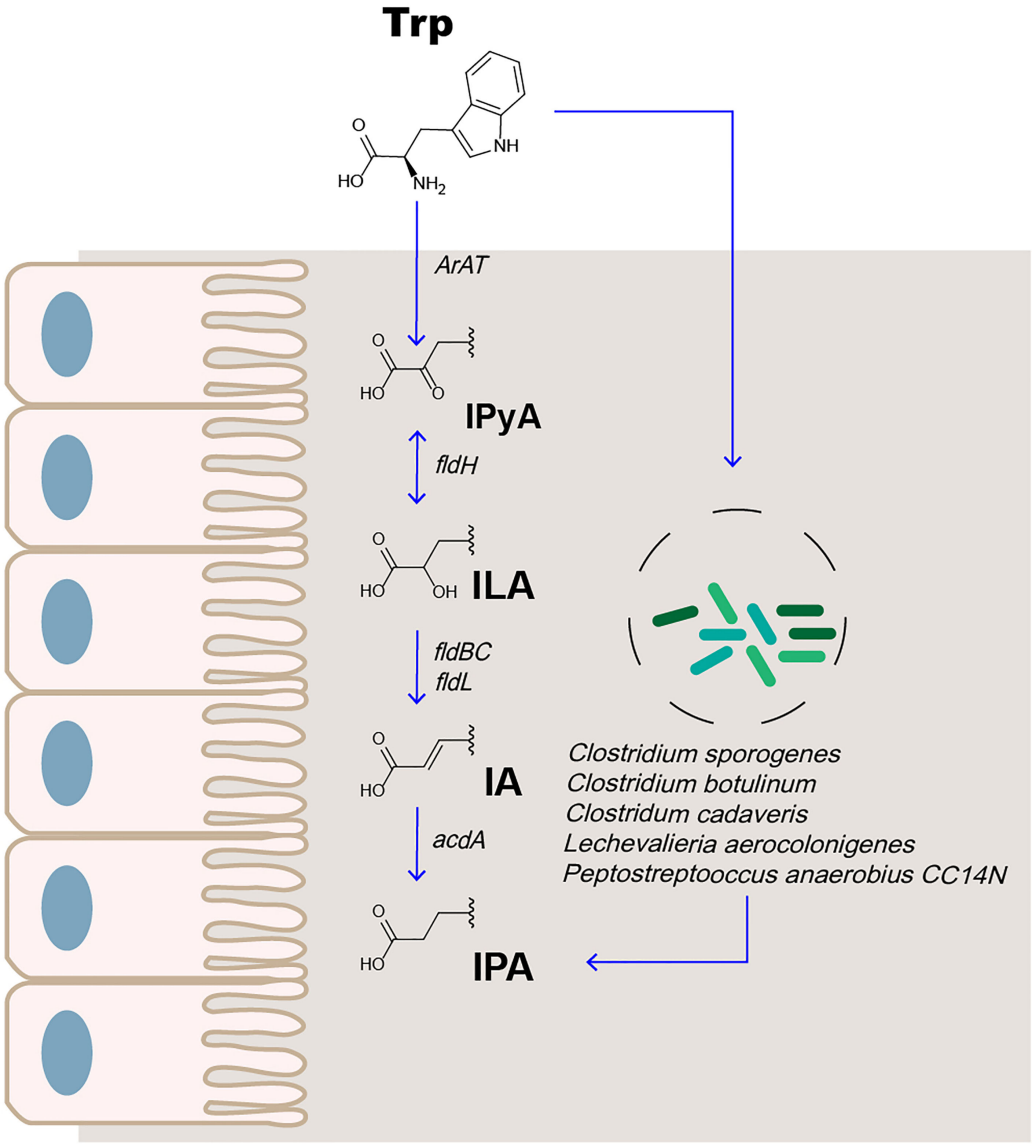
IPA is the metabolite of Trp in the intestine.

Trp is an essential amino acid from the host diet for use in protein synthesis (22). Trp is primarily metabolized through the 5-HT (23), canine uric acid (24), and intestinal microbial pathways. Indole-3-pyruvic acid (IPyA) is converted from Trp in the presence of an aromatic amino acid aminotransferase. IPyA is a precursor of indolelactic acid (ILA), and phenyllactate dehydrogenase is involved in this reduction reaction. Bacterial species containing phenyllactate dehydratase *(fldBC)* and its activator acyl-CoA ligase convert ILA to indoleacrylic acid (IA) through dehydration. IA can be further converted into IPA by acyl coenzyme A dehydrogenase, which is the final product of the reductive metabolism of Trp (**Figure 1**) (24–26). The most abundant metabolite of Trp in the intestine is indole, followed by indole-3-acetic acid and IPA (27, 28).

The metabolism of IPA in the body is affected by enzyme activity and intestinal microbes. Liquid chromatography–mass spectrometry (LC-MS) analysis was used to compare the plasma samples of sterile and conventional mice. The production of IPA was found to be entirely dependent on intestinal microbes. Colonization with *Clostridium sporogenes* and *Clostridium botulinum* can promote the concentration of IPA in the plasma (29, 30). Recently, the study found that, among 36 bacterial isolates cultured in Trp-containing medium, 4 (*Peptostreptococcus anaerobius CC14N* and 3 *Clostridium cadaveris* strains) were capable of producing IPA. Simultaneously, the presence of FLDC, a homologous cluster of the *fldBC* gene cluster, was found to be a reliable marker for IPA-producing bacteria (25). Other bacteria, such as *Lechevalieria aerocolonigenes*, can synthesize IPA through Trp deamination catalyzed by amino acid oxidase (31). Therefore, IPA is an important indicator of microbial metabolism.

In a study on the metabolic benefits of Trp, the Trp diet led to a decrease in mouse body weight (32); however, the mechanism was not elucidated. In another study, it was found that a diet supplemented with neomycin and Trp led to an increase in rat body weight, which was related to the significant change in the concentration of Trp-derived bacterial metabolites in the feces and blood. Further studies showed that the change in body weight increase was most relevant to the change in the concentration of the Trp metabolite IPA. The body weight gain in rats treated with IPA alone was two times lower than that in rats treated with the vehicle, suggesting that IPA might be an effector metabolite between a Trp-rich diet and lower body weight gain (33). Therefore, for the Trp-IPA metabolic pathway, the development of related probiotics, and the promotion of the production of IPA, we need to pay attention to the study of probiotics in the treatment of metabolic syndrome in the future.

## IPA Concentrations Affected by Dietary Intervention

Diet significantly impacts NAFLD, T2D, obesity, CVD, and metabolic disorders (34, 35). Therefore, we explored the relationship between IPA and diet. IPA was the metabolite most significantly and consistently related to both total carbohydrate and fiber intake (r = 0.28, *p* = 9.1 × 10^−5^ and r = 0.23, s = 0.001, respectively), including whole grain wheat, rye, and whole grain rye intake (12). In another study, 117 overweight adults were randomly divided into two groups. Based on the same diet, they were supplied with fried meat or not. The study found that the participants who consumed fried meat had higher lipopolysaccharide (LPS), tumor necrosis factor-α (TNF-α), interleukin-1β (IL-1β), and IL-10 levels (*p* < 0.05). Fried meat intake lowered microbial community richness and decreased *Lachnospiraceae* and *Flavonifractor* abundances while increasing *Dialister*, *Dorea*, and *Veillonella* abundances [*p* false discovery rate (FDR) < 0.05], which caused a significant decrease in the fecal metabolite IPA content (36).

In a diet study, 10 healthy participants were randomly fed a Western or Mediterranean diet for 4 days, and feces were collected for 16s RNA and metabolomics after 4 days. Different diets altered the intestinal flora structure. Simultaneously, IPA content in feces was significantly increased with the Mediterranean diet but decreased in the Western diet (37). This suggests that diet can affect the composition of intestinal microorganisms within a short time (38); however, the long-term effect and stability of the microbial structure are not apparent. Promoting the increase in IPA content may be an effective way to improve the metabolic benefits of the Mediterranean diet.

In the correlation experiment between 11 types of Trp metabolism levels and T2D events in the circulation of 9,180 participants from five cohorts, it was found that intake of fiber-rich foods, rather than protein/Trp-rich foods, and peripheral IPA content were positively correlated. Further research found that higher milk and fiber intake can improve the metabolism of Trp in the circulation of patients with T2D, but only in individuals with non-persistent genetic lactase (39). This suggests that diet can interfere with host–microbe interactions and affect the metabolism of Trp-IPA in the host. The effect of the metabolic benefits of a healthy diet is partly due to the promotion of IPA production in circulation.

## Role of IPA in Metabolic Diseases

We mainly discuss the relationship between IPA and various metabolic diseases, including obesity, T2D, NAFLD, and CVD, and focus on the potential connection between IPA and illness.

### IPA as a Potential Biomarker of Obesity and its Association With Inflammation

Obesity is a complex pathophysiological disease and one of the causes of metabolic syndrome, which is characterized by chronic low-grade inflammation. In 85 obese adults (average BMI = 40.48) and 42 non-obese control individuals (average BMI = 24.03), the serum IPA content was significantly lower in obese patients and was compared with BMI, serum high-sensitivity C-reactive protein (hsCRP), and high-sensitive interleukin 6 (hsIL-6), and the hsCRP and hsIL-6 levels were negatively correlated (40). This suggests that the indole metabolic pathway of Trp is affected in obese patients, which may be related to obesity-related systemic inflammation. However, in obese patients who underwent Roux-en-Y gastric bypass surgery (RYGB) operation, the level of IPA in the blood increased substantially 3 months post-surgery compared with 1 week post-surgery (40). This suggests that IPA content can be used as a marker for obesity. In future research, it will be necessary to perform correlation analyses between IPA and obesity-related complications to provide new diagnostic methods for invasive diagnosis of diseases and to predict obesity-related complications.

### IPA as a Potential Biomarker for Predicting the Risk of T2D

Current studies have found that IPA content is closely related to T2D and can predict the risk of T2D, distinguish different stages of T2D, and decrease with the improvement of T2D. IPA can be used as a biomarker of disease progression. When studying the brain–gut–microbiota characteristics of women with obesity and food addiction, a negative correlation was found between IPA in serum and food addiction (41). Bariatric surgery, such as RYGB, can improve T2D, obesity, NAFLD, and other metabolic diseases (42). In clinical studies, IPA content in the peripheral blood of obese patients with T2D was significantly lower than that in healthy participants. The IPA content in blood samples of these patients 3 months post-RYGB surgery was significantly higher than that in blood samples 1 week post-surgery (11).”XenoScan,” a metabolomics platform established by the University of California, Davis, used LC-MS to characterize a series of intestinal microflora metabolites and found that several metabolites, including IPA, could distinguish early T2D rats from rats 3 months after the onset of diabetes (43).

In a clinical trial, researchers used a non-targeted metabolomics approach to investigate whether serum metabolite profiles can predict the incidence of T2D in patients with impaired glucose tolerance. During the 15-year follow-up, patients with glucose tolerance who developed (n = 96) or did not develop (n = 104) T2D had lower and higher serum IPA levels, respectively. This suggests that higher serum IPA levels lead to a low risk of T2D (12). In a clinical study with a 7-year follow-up, it was verified that higher serum IPA levels were negatively correlated with the occurrence of T2D (OR [CI]: 0.86 [0.73–0.99], *p* = 0.04), directly correlated with insulin secretion (β = 0.10, *p* = 0.06), and negatively correlated with hsCRP when blood samples were collected (r = −0.22, *p* = 0.0001), and during follow-up visits (β = −0.19, *p* = 0.001). This suggests that IPA might be mediated by low-grade inflammation or enhance insulin sensitivity by protecting β-cell function to reduce the risk of T2D (21)

### IPA Reduces Lipotoxicity to Inhibit the Development of NAFLD

NAFLD manifests as liver fat accumulation, and the disease progresses to non-alcoholic steatohepatitis (NASH) or even hepatocellular carcinoma (HCC). Globally, the prevalence of NAFLD-related HCC may increase with an obesity epidemic (44).

IPA in the intestinal tract is absorbed by the intestinal epithelial cells and diffuses into the blood, which enters multiple target organs such as the liver after passing through the peripheral and portal circulation (30). This suggests that the liver may be a target organ for IPA biology. In 233 patients who underwent bariatric surgery and detailed liver histological examinations, the circulating IPA in patients with liver fibrosis was lower than that in those without fibrosis. Circulating IPA levels are also associated with the liver richness in genes that regulate hepatic stellate cell activation and liver fibrosis signaling. *In vitro* experiments have verified that IPA reduces the mRNA expression of fibrosis signaling markers such as *COL1A2*, *aSMA*, and *ITGA3* in LX-2 cells (13).

Cholesterol is considered the primary lipotoxic molecule among liver lipids in NASH development (45–47). Lipotoxicity promotes the progression of NAFLD to NASH, liver cirrhosis, and even liver cancer (48, 49). Depletion of IPA was noted in both hypercholesterolemia-fed HCC mice and in sterile mouse serum transplanted with hypercholesterolemia-fed HCC mouse feces. *In vitro* experiments showed that IPA could inhibit the accumulation of triglycerides (TG) in the cholesterol-induced human normal hepatocyte line LO2 and inhibit the proliferation of NASH-HCC cell lines (HKCI-2 and HKCI10). Therefore, the partial reason for cholesterol-induced lipotoxicity is the damage to tryptophan metabolism in microorganisms and the reduced serum IPA content, thereby promoting the development of NASH-HCC (50).

### IPA Improves CVD by Lowering Blood Lipid Levels

CVD is a serious cause of death due to metabolic diseases (51, 52). In a cohort study from an advanced atherosclerosis (n = 100) and gender- and age-matched control group (n = 20), the level of IPA in plasma metabolites of the advanced atherosclerosis group was significantly reduced (0.41 [0.27–0.90] μM vs. 0.22 [0.16–0.34] μM; *p* < 0.01). In a study of risk factors for atherosclerosis, IPA (OR, 0.27; 95% CI, 0.019–0.91; *p* = 0.02) was negatively correlated with advanced atherosclerosis (14). In mice experiments, oral administration of IPA significantly reduced high-fat diet (HFD)-induced body weight gain and reduced serum total cholesterol (TC), low-density lipoprotein cholesterol (LDL-c), and TG levels, showing sufficient anti-hyperlipidemic effects (16).

### IPA and Other Metabolic Diseases

In a 1-year follow-up study of patients with chronic kidney disease (CKD), the estimated glomerular filtration rate (eGFR) rapidly decreased by >20% (n = 10) and the control group (n = 10), and the eGFR decreased by <5%. It was found that IPA was the only metabolite that dropped significantly in eGFR rapidly decreased group of plasma. In cross-sectional clinical studies, it can also be found that the serum IPA content of the normal group was significantly higher than that of the CKD group (49.8 ± 15.9 vs. 34.7 ± 10.8 ng/ml; *p* < 0.01) (53). Intervention with IPA can also inhibit the gene expression of fibrosis and inflammation in proximal renal tubular cells induced by indophenol sulfate (54). In previous studies, oxidative stress was found to be associated with increased kidney damage (55), and IPA as a potent antioxidant may be an important bioprotective agent for CKD.

In another study, oral IPA supplementation reduced the systemic inflammation level in radiation-exposed mice, restored hematopoietic organs, relieved bone marrow suppression, and improved gastrointestinal function and epithelial integrity after irradiation, thereby exerting therapeutic effects on radiation toxicity (56). Supplementation with mouse probiotic *Clostridia* resulted in an increase in IPA production in the intestinal lumen and increased mitochondrial transcription factor A (Tfam) expression in osteoblasts by promoting Kdm6b/Jmjd3 histone demethylase, thereby inhibiting the epigenetic methylation of H3K27me3 at the Tfam promoter from preventing pathological bone loss in obese mice induced by a HFD (57). IPA is neuroprotective as a potent hydroxyl radical scavenger (58). IPA inhibits β-amyloid fibril formation, a potent neuroprotective agent, and is a potential drug for the treatment of Alzheimer’s disease (59). IPA also exhibits protective effects against streptozotocin-induced diabetic peripheral neuropathy in rats and high-glucose-induced neurotoxicity in neural 2a cells (60).

## Mechanisms of IPA Action on Metabolic Diseases

As mentioned above, IPA contributes to various metabolic diseases, and its mechanism is complex. It may be involved in the physiological and pathological processes of the disease through different pathways. Therefore, we comprehensively analyzed the mechanism of IPA in terms of glucose metabolism, insulin resistance, lipid synthesis, inflammatory reactions, and the intestinal microenvironment.

### IPA Can Improve Blood Glucose and Increase Insulin Sensitivity

Impaired glucose tolerance and insulin tolerance are also pathogenic factors in metabolic syndrome. Rats fed a diet rich in IPA had improved glucose metabolism and significantly reduced HOMA index of fasting blood glucose, insulin, and insulin resistance (15).

Cognitive decline is a complication of T2D, and intermittent fasting (IF) is a dietary intervention used to alleviate the symptoms of T2D. In research of its mechanism, IF was found to improve cognition through the microorganism–metabolite–brain axis. Among the metabolites affected by IF, the complementary metabolite IPA showed similar results with IF. In db/db mice, IPA was found to improve cognitive function and insulin sensitivity, enhance mitochondrial biogenesis, and protect the ultrastructure of synapses (61). This may be related to IPA as an antioxidant, preventing neuronal death induced by amylin and β-amyloids and restoring mitochondrial function (62, 63).

First, the protective effect of serum IPA in T2D may be achieved through its efficacy in regulating the secretion of incretin, particularly glucagon-like peptide (GLP)-1 release by intestinal endocrine L cells (64). GLP-1 inhibits the occurrence of T2D by reducing B-cell apoptosis and increasing cell proliferation and regeneration (65)

Second, as a strong oxidant (62), IPA can protect β cells from damage related to metabolism and oxidative stress, and possibly from the accumulation of amyloid (66). These results suggest that IPA may be a promising candidate for the treatment of insulin-resistant metabolic disorders, including T2D.

### IPA Inhibits Liver Lipid Synthesis and Inflammatory Factors

IPA intervention can improve NASH model mice induced by a HFD through intestinal microenvironment homeostasis (17). In an *in vitro* experiment, supplementation with oleic acid (OA; 100 μM) resulted in significant accumulation of TG in a human hepatocarcinoma cell line of HepG2 cells, and IPA treatment significantly reduced OA-induced TG accumulation in a dose-dependent manner (10, 25, and 50 µM). Further research showed that IPA dose-dependently reduced the transcription of essential genes involved in fatty acid (*Srebp1-c* and *Fas*) and cholesterol biosynthesis (*Srebp2* and *Hmgr*) in HepG2 cells (16).

In addition to inhibiting lipid synthesis in the liver, IPA intervention could inhibit the expression levels of pro-inflammatory cytokines such as TNF-α, IL-1β, and IL-6 in the liver of NASH rats induced by a HFD. In an *in vitro* LPS-induced mouse macrophage model, IPA also inhibited nuclear factor kappa B) NF-κB signaling, p65 phosphorylation, and the expression of NF-κB downstream target genes in a dose-dependent manner (17).

Excess free Fe(3+) and was also found to cause oxidative damage, which deteriorates NAFLD to NASH. In an *in vitro* experiment, FeCl(3+) (0.2 mM) was used to induce the isolated rat liver microsomes to simulate the oxidative damage model and then incubated with IPA. It was found that co-incubated IPA (concentrations of 10, 3, 2, and 1 mM) can prevent the decrease in cell membrane fluidity caused by Fe(3+). The increase in lipid peroxidation caused by Fe(3+) was only inhibited after incubation with the highest concentration (10 mM) of IPA (67). Moreover, IPA at a concentration of 5 mM was able to inhibit lipid peroxidation damage in hamster testes caused by iron ions (68). This suggests that IPA can act as an effective free radical scavenger to prevent iron-induced oxidative damage to cell membranes. The antioxidant effect of IPA is concentration dependent, which also explains the protective effect of high concentrations of IPA on the periphery of the body.

### IPA Can Correct Intestinal Microbial Disorders

IPA maintains the stability of the intestinal microenvironment. Its primary mechanism is to correct the disordered intestinal microflora, repair the intestinal barrier, and inhibit the intestinal immune response.

The intestinal microbial structure can affect the host’s absorption of dietary monosaccharides and lipids, promoting the accumulation of TG in the adipose tissue and liver and causing metabolic disease (69, 70). An imbalance of intestinal microbes affects the TLR9- and TLR4-related inflammatory pathways in the liver (71). In multi-ethnic cohort studies, intestinal microbial α diversity was generally low in patients with metabolic diseases (72). In a clinical cohort study of 1,018 middle-aged women from TwinsUK, the relationship between serum IPA levels and gut microbial genes was evaluated, and a positive correlation between microbiota alpha diversity and serum IPA content was found (Shannon diversity: β [Shannon diversity: beta (95% CI] = 0.19 [0.13; 0.25], *p* = 6.41 × 10^−10^) (73).

In an 8-week NAFLD rat model induced by a HFD, IPA (20 mg/kg) was administered to rats for the 8-week experiment. The 16s rRNA method was used to detect rat feces, and principal coordinate and non-metric multidimensional scale analyses showed that the intestinal microbes of rats in the IPA administration group were significantly different from those in the model group. This suggests that IPA administration can improve the overall structure of the intestinal microbes in NAFLD rats. An increase and decrease in the abundance of *Firmicutes* and *Bacteroidetes*, respectively, were biomarkers of obesity (74, 75). Based on this feature, the authors analyzed the intestinal microbial composition, and an HFD was found to increase the ratio of *Firmicutes* to *Bacteroidetes*, which could be reversed by IPA treatment. The abundance of the two potential pathogenic bacteria *Bacteroides* and *Streptococcus (* 76) was increased by an HFD and decreased by IPA treatment. It has also been reported that the abundance of the genus *Parasutterella (* 77) associated with chronic intestinal inflammation was reduced by IPA treatment. In addition, the abundance of the two genera *Oscillibacter* and *Odoribacter*, which are important for maintaining intestinal homeostasis, were reduced in the HFD-fed group (78) and restored in the IPA group (17). In addition, IPA supplementation can inhibit the growth of *Mycobacterium tuberculosis* by blocking the synthesis of Trp in *M. tuberculosis* through the catalytic step of TrpE, thereby exerting an anti-tubercular effect (79).

### IPA Maintains the Intestinal Barrier

Increased intestinal permeability and abnormal intestinal tight junctions caused by ecological imbalance are frequently observed in patients with metabolic diseases (80). Intestinal ecological imbalance leads to an increase in LPS and bile acid production, which is related to whole-body low-grade inflammation (81).

Treatment of HFD-fed mice with IPA reduced intestinal permeability (decreased circulating FITC-dextran) and reduced circulating LPS levels. *In vitro*, researchers used monolayers of T84 cells incubated with the pro-inflammatory cytokines interferon-γ (IFN-γ) and TNF-α. IPA was found to reduce the permeability of monolayers through an FITC-dextran permeability experiment (82).

The ratio of villi to crypts in the ileum of HFD-fed rats was reduced (83), and the villi height was restored by IPA treatment, which also promoted the protein expressions of zonula occludens-1 (ZO-1), occludin, and tight junction proteins in the rat ileum (17). The end of the afferent neurons of the vagus nerve is located in the intestinal mucosa, and the increase in LPS changes the afferent signals of the vagus nerve and reduces the satiety induced by cholecystokinin, thus promoting appetite and leading to obesity (84). Thus, IPA can inhibit appetite by inhibiting LPS levels in the plasma.

The Caco-2/HT29 co-culture model was used to evaluate the effect of IPA on the intestinal barrier and explore its potential mechanism. Studies have shown that IPA increases transepithelial resistance and decreases paracellular permeability. Simultaneously, IPA enhances the mucus barrier by increasing the expression of mucins MUC2 and MUC4 and the goblet cell secretion products TFF3 and RELMβ. In addition, IPA reduces the expression of inflammatory factors in LPS-induced Caco-2/HT29 cells. These findings provide a new perspective for the intestinal microbial metabolite of Trp to improve the intestinal barrier (85). SLC2A5 (GLUT5-encoded) is the leading fructose absorption transporter in the kidneys, small intestine, and proximal tubules, and its overexpression causes metabolic syndrome by increasing fructose intake (86). Expression of the fructose transporter *SLC2A5* mRNA was increased in IFN-γ-induced intestinal epithelial T84 cells, and IPA intervention reversed *SLC2A5* mRNA expression (11).

### IPA Suppresses Intestinal Immune Response

Impaired intestinal barrier function and increased leakage of intestinal-derived antigens may lead to visceral lipid deposition and metabolic dysfunction (87). Serum IPA was reported to be decreased by approximately 60% in patients with active inflammatory bowel disease compared with that in healthy controls. During the recovery period of inflammatory bowel disease, the level of IPA in the serum gradually recovered (27, 88).

Administration of IPA showed significant induction of IL-10 receptor protein 1 expression in cultured intestinal epithelial cells T84 (27), based on a close correlation between epithelial IL-10 receptors and the maintenance and recovery of epithelial barrier function (89), which further supported the role of IPA in the maintenance of intestinal immunity.

Recent studies have suggested that IPA is an endogenous ligand for intestinal PXR. IPA induces the transcription of PXR target genes *Mdr1*, *Cyp3a11*, and *Ugt1a1* mRNA *in vivo (* 82). However, IPA alone is a weak human PXR ligand (82). Inoculated *Clostridium sporogenes* in germ-free mice accompanied with L-Trp-supplemented diets promoted the production of IPA to protect mice from dextran sulfate sodium-induced colitis through the PXR pathway (82). Studies have also confirmed that IPA improves intestinal permeability (FITC-dextran permeability) in a colitis (indomethacin-induced) mouse model with intestinal barrier defects. Intestinal *TNF-α* mRNA expression and p38-MAPK protein phosphorylation were inhibited, while in PXR-deficient (Nr1i2^−/−^) mice, the benefits of IPA were inhibited, suggesting that IPA improved the intestinal barrier *via* the PXR pathway (82). Furthermore, IPA can modulate vascular function by modulating PXR activity, and IPA exposure reduces the vasodilatory responses of nitric oxide-mediated muscarinic and protease-activated receptor 2-stimulated mouse aortic tissue (90).

In another study, IPA was shown to be an agonist of the aromatic meridian receptor (Ahr) of a commensal bacterial product (91), and Ahr activation was beneficial to the maintenance of intestinal homeostasis and the regulation of immunity (92, 93). Therefore, these studies indicated that promoting IPA formation by bacteria or the direct administration of IPA is beneficial for inflammatory bowel disease.

## Conclusion

**Figure 2:**
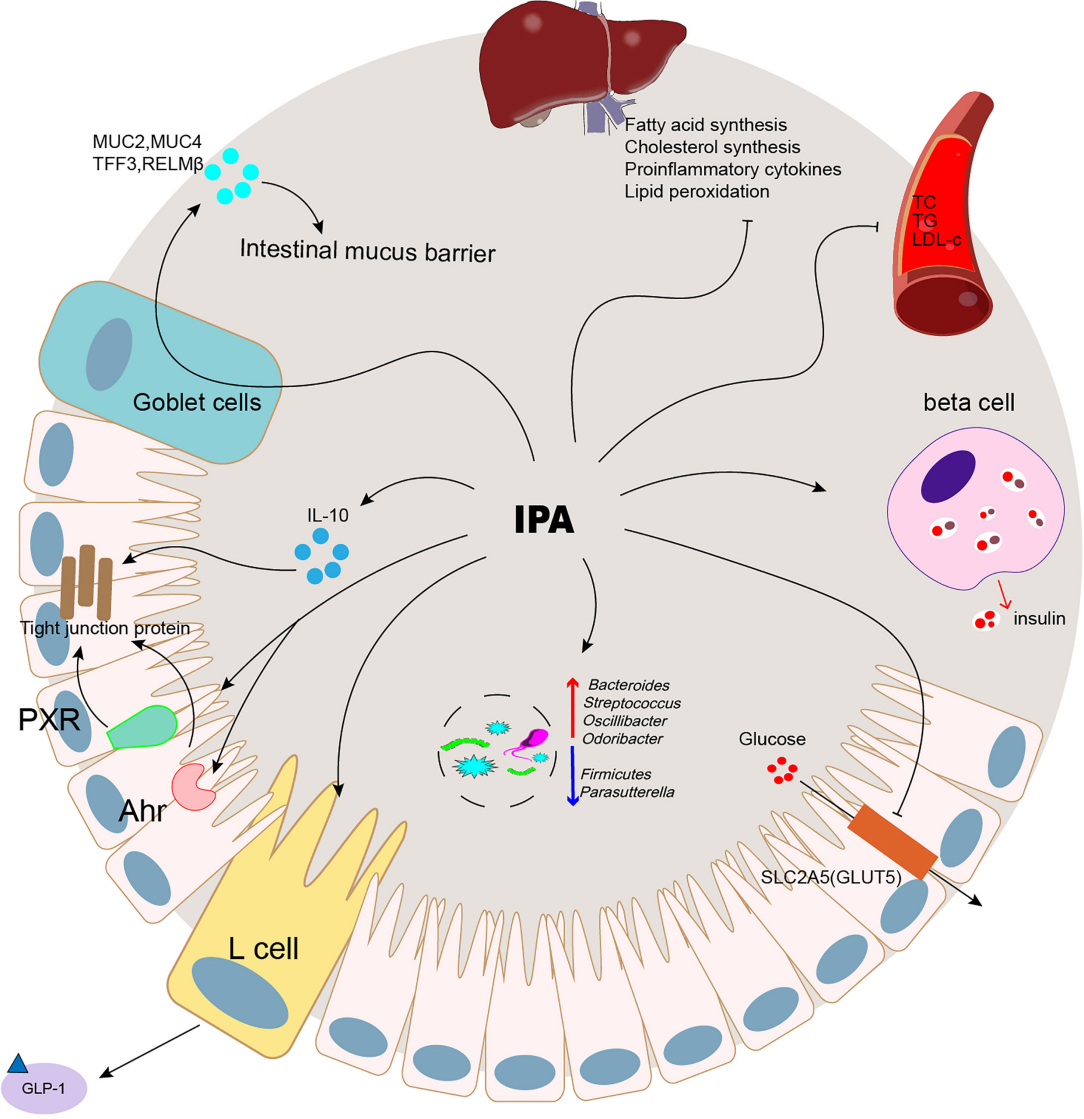
IPA was correlated with the occurrence and development of metabolic diseases.

The intestinal microflora is a diverse microbial community that encodes functional genes several orders of magnitude higher than the human genome and can regulate human health (94). With the development of metabolomics, intestinal microbial metabolites play an increasingly important role in regulating host health and disease; however, the disturbance of metabolites is related to multiple chronic diseases (95). Therefore, research on bacteria-derived metabolites offers the possibility of personalized medicine for chronic diseases with complex pathogenesis. The study found that IPA, a metabolite produced by Trp under the action of intestinal microbes, was correlated with the occurrence and development of metabolic diseases (**Figure 2**). Metabolic diseases such as obesity, T2D, NAFLD, and CVD have been reported. The IPA content in the peripheral region was significantly consumed. After 3 months of bariatric surgery, it recovered, suggesting that IPA might be a potential biomarker for metabolic diseases. However, further studies have shown that IPA could be a potential biomarker of metabolic illnesses (**Table 1**). The intervention with IPA reduced the body weight and peripheral fat content; improved insulin resistance, liver lipid deposition, and peripheral blood lipid content; and maintained intestinal homeostasis, thereby improving metabolic syndrome (**Table 2**). The metabolic benefit mechanism of IPA may be predominantly related to its strong oxidant effect, which has an excellent antagonistic effect on chronic inflammation caused by metabolic diseases. Moreover, as a bacterial-derived metabolite (**Table 3**), IPA exerts its beneficial effects in regulating intestinal immune responses through Ahr and PXR ligand. Intestinal bacteria play an important role in the pathogenesis of metabolic diseases. In future research, we need to pay attention to the secondary metabolites produced by the interaction between IPA and the bacterial flora and its remote target organs to further study the mechanism of IPA.

**Table 1:** The relationship between IPA and metabolic diseases.

Disease	Research object	Clinical trials number	Study population nation	Sample	Detection method	Main research results	Reference
Liver fibrosis	A total of 233 patients (BMI 43.1 *±* 5.4 kg/m^2^) undergoing bariatric surgery with detailed liver histology were included. Normal liver (n = 79), Simple steatosis (n = 40), NASH (n = 45)	NA	Finland (Europe)	Serum	LC-MS	IPA levels were decreased in liver fibrosis compared to those without fibrosis (*p* = 0.039 for all participants; *p* = 0.013 for 153 individuals without T2D); IPA levels negatively correlated with lobular inflammation (*p* = 0.039) and fibrosis (*p* = 0.039); IPA levels negatively correlated with fibrosis signaling genes, including *ITGA3*, *ITGAV*, *LAMC3*, and *COL1A2* mRNA	(13)
T2D	Prospective analysis of 11 circulating Trp metabolites and T2D incidence; up to 9180 participants from 5 cohorts by meta-analysis	NA	Diverse racial/ethnic backgrounds (USA)	Serum	LC-MS	IPA levels positively associated with fiber-rich foods (*p* = 7.3×10^−60^); IPA negatively associated with T2D incidence, (Spearman’s r = −0.05 to 0.06); IPA showed a potential causal relationship with T2D (genetic causality proportion = 76%, *p* = 1.6×10^−24^)	(39)
T2D	Total 415 diabetes participants lifestyle (n = 209); control groups (n = 206)	NCT00518167	Finland (Europe)	Serum	HPLC-QQQ-MS/MS	IPA levels inversely associated with incidence of diabetes during the mean 7-year follow-up (odds ratio [confidence interval]: 0.86 [0.73–0.99], *p* = 0.04); positively correlated with insulin secretion (DI30) during the mean 7 years (β = 0.10, *p* = 0.06; positively correlated with dietary fiber, r = 0.24, *p* = 1 × 10^−6^); inversely associated with serum hsCRP levels (r = −0.22, *p* = 0.0001); inversely associated with BMI (*p* = 0.001)	(21)
T2D	Two groups of individuals who took part in the Finnish Diabetes Prevention Study Those who either early developed T2D early (n = 96) or did not develop T2D (n = 104) within the 15-year follow-up	NCT00518167	Finland, Sweden (Europe)	Serum	LC-MS	IPA levels inversely associated with T2D incidence (OR: 0.80 [0.70, 0.93], *p* = 0.003); positively correlated with insulin secretion (β = 0.25 [0.06–0.44], *p* = 0.011); inversely associated with high hsCRP levels (r = -0.23, *p* = 0.006); high IPA level inversely associated with the likelihood of developing T2D during the 5-year follow-up (OR: 0.31 [0.12– 0.76], *p* = 0.01)	(12)
Advanced atherosclerosis	Advanced atherosclerosis cohort (n = 100); the control cohort (n = 22) were age- and sex-matched participants	NA	USA	Serum	LC-MS	IPA content decreases in advanced atherosclerosis and carotid stenosis subgroups. IPA levels inversely associated with advanced atherosclerosis incidence (OR, 0.27; 95% CI, 0.019–0.91; *p* = 02)	(14)
Obesity	obese adults (n = 85, BMI = 40.48); non-obese controls (n = 42, BMI = 24.03)	Registration numbers 2010/36 and 2016/40 for obese and non-obese participants, respectively	France (Europe)	Serum	UHPLC-ESI-MS/MS	IPA content decreases in obesity (F[1,122] =13.89*, p*<0.001); IPA levels inversely associated with BMI (data not shown); inversely associated with serum levels Of hsCRP (β = −0.268 0.261, *p* < 0.05) hsIL-6 levels (β = −0.244, *P* < 0.05)	(40)
Obesity	A total of 117 overweight (BMI > 24 kg/m^2^) adults were randomized into two groups. One group was provided fried meat four times per week (n = 59); one group of 58 participants had no fried meat intake (n = 58).	ChiCTR1900028562	China (Asia)	Fecal	UPLC-MS/MS	IPA content decreases in fried meat group (*p* FDR < 0.05); IPA levels inversely associated with insulin resistance index (r = 0.243); inversely associated with serum LPS levels (r = 0.243); IPA levels inversely associated with serum TNF-a levels (r = 0.436)	(36)
Obesity	Food addiction (n = 19,BMI = 35.6); No food addiction (n = 86)	IRB # 16–000187	USA	Fecal	Mass spectroscopy	IPA was inversely associated with food addiction in patients with obesity (Cohen’s d = 0.74, *p* = 0.045); inversely associated with abundance of genus Prevotella; positively correlated with abundance of *Akkermansia muciniphila* and *Bacteroides*	(41)
Obese T2D	Lean (n = 7); obese T2D participants either before (n = 9) or after RYGB surgery (1 week post-surgery [n=9]; 3 months post-surgery [n=7])	NA	NA	Serum	LC-MS	IPA content decreased in fried meat group obese T2D; unchanged 1 week after RYGB surgery; increased 3 months after RYGB surgery	(11)
Chronic kidney disease	The estimated glomerular filtration rate (eGFR) rapid decline 20% group (n = 10) vs. control group (n = 10) was defined as having a yearly eGFR decline < 5%; the CKD group (n = 140) vs. the normal group (n = 144).	IRB no. 100-2243A3	China	Serum	NA	IPA content decreased in the CKD group; IPA content decreased in patients with rapid decline 20% group	(53)
UC	Healthy controls (n = 20); participants with active ulcerative colitis (UC; n = 15); participants with UC in remission (n = 20)	NA	NA	Serum	EC-HPLC	Serum IPA was decreased by approximately 60% in participants with active UC compared to healthy controls (*p* < 0.05); IPA content returned to normal in participants with UC in remission	(27)

NA, Not Available.

**Table 2:** Benefits of IPA in metabolic diseases.

Disease	Modeling	IPA concentration	Result	Mechanism	References
Liver fibrosis	LX-2 cell co-treatment with TGF-β1	100 µM	IPA treatment with 100 µM of IPA also significantly reduced LX-2 cell migration	IPA treatment reduced activation of LX-2 cells stimulated by TGF-β1; reduced hepatic stellate cell activation gene expression of *COL1A2, aSMA, ITGA3* mRNA	(13)
T2D	Male Sprague–Dawley rats (diet not shown)	Mean intake 27.3 mg/kg/day	IPA was associated with a reduction in fasting blood glucose concentration by 0.42 mM (95% CI: 0.11–0.73; t22 = 2.78; *p* = 0.01); IPA treatment reduced plasma insulin level (t19 = 2.26; *p* = 04) and the HOMA index (t19 = 2.46; *p* = 02)	NA	(15)
T2D cognitive decline	db/db mice fed with regular chow and pure water	Mice were intraperitoneally injected with IPA (10 mg kg/day) for 14 days	IPA treatment significantly attenuated cognitive deficits in diabetic mice; improved insulin sensitivity; enhanced mitochondrial biogenesis, and protected the ultrastructure of synapses.	IPA has been reported to protect against Aβ-induced neuronal death and restore mitochondrial function	(61)
Obesity	High-fat diet (HFD)-fed mice	20 mg kg^−1^ po. for 4 days	IPA treatment did not change body weight; significantly attenuated intestinal permeability; and reduced LPS levels.	IPA treatment reduced protein expression of SLC2A5 (GLUT5, facilitated fructose transporter) and ALDOB (fructose-1,6-bisphosphate aldolase glycolytic enzyme) in T84 cells	(40)
NAFLD	Sprague–Dawley rats; rats were fed a standard chow diet or a HFD	Gavage with IPA (20 mg/kg/day) for 8 weeks	IPA treatment modulated the microbiota composition in the gut and inhibited microbial dysbiosis in rats fed a HFD.	IPA induced the expression of tight junction proteins, such as ZO-1 and occludin, and maintained intestinal epithelium homeostasis, leading to a reduction in plasma endotoxin levels. IPA inhibited NF-κB signaling and reduced the levels of proinflammatory cytokines, such as *TNFα, IL-1β*, and *IL-6*, in response to endotoxin in macrophages to repress hepatic inflammation and liver injury	(17)
NASH-HCC	Cholesterol-induced hepatocyte cell line LO2, and NASH–HCC cell lines HKCl-2 and HKCl-10	IPA (10 μM,100 μM)	IPA treatment suppressed cholesterol-induced lipid accumulation in LO2 cells, and cell proliferation in NAFLD-HCC cell lines (HKCI-2 and HKCI-10).	NA	(50)
HCC	Rat hepatic microsomal membrane incubated with FeCl(3) (0.2 mM), ADP (1.7 mM), and NADPH (0.2 mM) to induce oxidative damage	IPA (10, 3, 2, 1, 0.3, 0.1, 0.01 or 0.001 mM)	IPA may be used as a pharmacological agent to protect against iron-induced oxidative damage to membranes and, potentially, against carcinogenesis.	IPA, when used in concentrations of 10, 3, or 2 mM, increased membrane fluidity; IPA at concentrations of 10, 3, 2, or 1 mM completely prevented a decrease in membrane fluidity due to Fe(3+); the enhanced lipid peroxidation due to Fe(3+) was prevented by IPA only at the highest concentration (10 mM)	(67)
Hyperlipidemia	Male and female ICR mice	Orally administered IPA (100 mg/kg) for 60 days	IPA treatment significantly reduced the body weight gain in mice; decreased serum levels of TC, LDL-c, and TG.	IPA dose-dependently decreased the transcription of the key genes involved in fatty acid (*SREBP1c* and *FAS*) and cholesterol biosynthesis (*SREBP2* and *HMGR)*	(16)
IBD	Nr1i2^+/+^ and Nr1i2^−/−^ mice using an inflammation-based barrier defect (indomethacin) model	Mice were gavaged with 10, 20, and 40 mg/kg IPA for 4 days	IPA treatment significantly reduced FITC dextran permeability in Nr1i2^+/+^ mice, but not in Nr1i2^−/−^, mice; IPA notably decreased TNF-a mRNA expression more in the Nr1i2^+/+^ mice (3.73-fold) intestinal epithelium relative to Nr1i2^−/−^ mice (1.72-fold)	IPA Protects against indomethacin-induced intestinal injury *via* PXR and TLR4	(82)
IBD	C57BL/6 mice were administered 2.5% (wt/vol) dextran sodium sulfate (DSS)	IPA 0.1 mg/ml was administered to water for 9 days	Serum indole and IPA levels were significantly decreased in actively colitic animals (*p* < 0.05)	DSS colitic mice displayed significantly lower levels of IPA (*p* < 0.01); IPA-treated animals displayed significantly less reduction in colon length (*p* < 0.05); IPA-treated mice had decreased colonic tissue cytokine levels: IFN-γ (*p* < 0.05), TNF-α (*p* < 0.01), IL-1β (*p* < 0.05) mRNA	(27)

NA, Not Available.

**Table 3:** Microorganisms that produce IPA.

Microorganisms	Relation	References
*Escherichia coli*	Produce	(96)
*E. coli, Bacillus* spp., and *Clostridium* spp.	Produce	(97)
*Clostridium sporogenes*(ATCC 15579)	Produce	(30)
*Akkermansia* and *Clostridium* XIVa	Correlation exists	(16)
*Peptostreptococcus anaerobius* CC14N *Clostridium cadaveris* CC88A *Clostridium cadaveris* CC44 001G *Clostridium cadaveris* CC40 001C	Produce	(25)
*Clostridioides caloritolerans* *Clostridioides. Botulinum*	Produce	(29)

However, the beneficial effects of IPA are all based on the HFD-induced NAFLD model mice, and the opposite result has been found in other models. In CCL4-induced liver fibrosis model mice, IPA aggravated CCL4-induced liver fibrosis injury through transforming growth factor-β1 (TGF-β1) and the Smad signaling pathway (98). Therefore, multiple models are required to verify the potential beneficial effects of IPA on metabolic diseases. At present, the therapeutic effects of IPA are primarily concentrated in basic animal experiments, and no clinical experiments have been performed. Therefore, an in-depth study on the toxicity and safe use of IPA is necessary to provide a sufficient theoretical basis for the development and utilization of IPA.

Furthermore, to fully exploit the potential of the intestinal microbiota in disease prevention, we need to understand in greater depth how dietary components and host genetics affect IPA production. Finally, these findings are converted into clinical practice and developed into clinical methods that can be widely used to predict the prognosis and outcome of diseases and even have diagnostic effects on some metabolic disorders. While leveraging metabolomics poses significant challenges in promoting human health, past studies have demonstrated that certain metabolites have considerable potential for the treatment of human diseases.

## Author Contributions

BZ and MJ: writing—original draft. These authors have contributed equally to this work. YS and JZ: writing—review and editing. WD and JS: funding acquisition. All authors contributed to the article and approved the submitted version.

## Funding

This work was supported by the National Natural Science Foundation of China (No. 81570524); Key Medical Disciplines of Hangzhou; Zhejiang Province Basic Public Welfare Research Program Project "Relationship between cognitive impairment and gut microbial dysbiosis in patients with non-alcoholic fatty liver disease" (GF20H030035).

## Conflict of Interest

The authors declare that the research was conducted in the absence of any commercial or financial relationships that could be construed as a potential conflict of interest.

## Publisher’s Note

All claims expressed in this article are solely those of the authors and do not necessarily represent those of their affiliated organizations, or those of the publisher, the editors and the reviewers. Any product that may be evaluated in this article, or claim that may be made by its manufacturer, is not guaranteed or endorsed by the publisher.
